# Psychiatric nurses’ knowledge and practices towards patients’ tobacco-related habits in mental health hospitals in Greece

**DOI:** 10.1186/1617-9625-12-S1-A27

**Published:** 2014-06-06

**Authors:** Evmorfia Koukia, Theodore Stathopoulos

**Affiliations:** 1 Psychiatric Nursing, Faculty of Nursing, University of Athens, Athens, 157-73, Greece

## Background

The aim of this study was to identify (a) nurses’ knowledge towards patients’ smoking habits (b) nurses’ beliefs towards psychiatric patients’ smoking practices and (c) nurses’ attitudes and practices.

## Materials and methods

A questionnaire based study was contacted among psychiatric nurses working on two major psychiatric hospitals. The total sample consisted of 125 psychiatric nurses (4-year education in a faculty of nursing of Technological Educational Institute) which represents the 48% of licensed nurses working full-time.

## Results

Various practices were noted among nurses concerning the assessment of patients’ smoking history, passive smoking, smoking habits and cessation plans. The majority of nurses (56.0% yes, 28.0% sometimes) noted that psychiatric patient should be handled differently. They stated that smoking cessation may exacerbate psychiatric symptoms (38.0% yes, 62.0% sometimes) and may lead to an illness relapse (46.0% yes, 44.0% sometimes). Nurses had some knowledge about the health effects of smoking and they feel responsible to help patient quit smoking.

## Conclusions

To our knowledge this is the first attempt to describe tobacco-related knowledge and practices among psychiatric nurses in Greece. The findings indicated that half of psychiatric nurses smoke in their work environment and are against the application of the anti-smoking law in psychiatric hospitals. They believe that psychiatric patients should be handled different from other patients even though they are aware of the dangers of smoking.

